# African swine fever control in the context of endemic persistence

**DOI:** 10.3389/fvets.2026.1891840

**Published:** 2026-09-10

**Authors:** Susanne Küker, Dirk U. Pfeiffer, Ulrich Kihm, Patrik Buholzer

**Affiliations:** 1 SAFOSO AG, Liebefeld, Switzerland; 2 TAFS Forum, Liebefeld, Switzerland; 3 Department of Infectious Diseases and Public Health, City University of Hong Kong, Kowloon, Hong Kong SAR, China; 4 Department of Pathobiology and Population Sciences, Royal Veterinary College, London, United Kingdom

**Keywords:** African swine fever, ASF, control, surveillance, vaccines

## Abstract

Increasing evidence suggests that, in some regions with prolonged ASFV circulation, ASF epidemiology is increasingly shaped by endemic persistence and ongoing viral diversification. Emerging attenuated and recombinant variants, together with evolving control strategies and increasing interest in vaccination, may alter transmission dynamics and the selective environment for the ASF-virus. At the same time, biological constraints associated with vaccine development and heterogeneity in surveillance systems may complicate disease detection and interpretation of emerging epidemiological patterns. We propose that ASF should increasingly be viewed as a long-term adaptive management challenge requiring coordinated approaches that integrate vaccination, surveillance, and evolutionary considerations across diverse production systems.

## 1 Introduction

African swine fever (ASF) represents one of the most consequential transboundary animal diseases of the 21st century. While ASF poses no zoonotic risk to humans (1), it has profound and destabilizing effects on global pork production systems, international trade frameworks, and food security governance (2, 3). Consequently, classical disease control mechanisms such as stamping-out policies, movement restrictions, trade restrictions, have been put in place globally to contain outbreaks and prevent cross-border spread (4). However, while acute and highly fatal outbreaks continue to occur in several regions, growing evidence suggests that, in some areas with prolonged ASFV circulation, the epidemiological landscape is increasingly also shaped by endemic persistence and ongoing viral evolution (5, 6). Endemic persistence itself is not a new phenomenon in ASF, particularly in parts of sub-Saharan Africa. However, its increasing relevance in newly affected regions coincides with viral diversification, changing control practices, the emerging use of live attenuated vaccines, and heterogeneous surveillance capacities. This Perspective examines how the convergence of these developments may alter ASF epidemiology and argues that their implications for disease control need to be considered collectively rather than as isolated challenges.

## 2 From acute outbreaks to endemic persistence

Following introduction into previously unaffected areas, ASF typically presents as an acute, highly virulent disease in domestic pigs with high mortality (7, 8). In contrast, regions with prolonged virus circulation, such as sub-Saharan Africa, the Iberian Peninsula, and Sardinia and, more recently, in parts of Asia following sustained genotype II circulation, where reports of attenuated variants and atypical clinical presentations have emerged, have been characterized by atypical, chronic, or subclinical disease presentations, consistent with gradual adaptation in host–virus dynamics (9–12). In these settings, infections often show milder or prolonged clinical courses, allowing infected animals to survive longer and potentially contribute to sustained transmission. As epidemic spread transitions into sustained endemic circulation, both transmission dynamics and the selective environment change (5). Continued virus circulation in large and densely connected pig populations promotes genetic diversification, increasing the likelihood that variants with altered or reduced virulence emerge (5, 9). After the introduction of ASFV into Europe in 2007 and East Asia in 2018, similar developments have been observed (5, 13). In several affected regions, the initial high-mortality phase was followed by recurrent outbreaks with sustained circulation and increasing genetic and phenotypic heterogeneity, including attenuated and recombinant field variants (12, 14–16).

While prolonged ASFV circulation and less acute disease presentations have long been recognized in endemic regions, particularly in parts of sub-Saharan Africa, their emergence in more recently affected regions occurs within different epidemiological contexts, including highly connected pig production systems and extensive transboundary movements. As a consequence, clinical signs may become less pronounced or less specific, reducing the reliability of overt disease as an early warning indicator and complicating timely detection and response. In systems where control measures are triggered primarily by the recognition of severe clinical signs, this shift has significant operational implications. In particular, surveillance frameworks that rely heavily on passive reporting of clinically apparent disease may become less sensitive to ongoing transmission.

## 3 Control interventions as selective pressure

Control interventions inherently shape pathogen evolution. Additional selective pressure may arise when intervention thresholds are tied to clinical severity rather than laboratory-confirmed infection status (17). In several Asian settings, particularly following the widespread establishment of ASF, control strategies have shifted from whole-herd depopulation toward partial depopulation or so-called “tooth extraction” approaches (17–20). Reports from Vietnam demonstrate that such approaches are often implemented in the absence of systematic laboratory confirmation and rely predominantly on the visibility and progression of clinical signs to trigger removal decisions (17). As a result, ASFV strains that cause acute, severe disease may be removed early, whereas those producing subclinical, protracted or atypical disease phenotypes gain a relative fitness advantage. Consequently, infected animals with delayed, mild or atypical clinical courses may remain within the population for prolonged periods, continuing to shed virus and contribute to onward transmission (20). These observations illustrate a core epidemiological principle: control interventions do not merely suppress disease, they define the selective environment in which pathogens survive and evolve. If intervention thresholds are tied to clinical severity rather than infection status, control measures may unintentionally select for pathogens that are less clinically apparent, more persistent and ultimately more difficult to eliminate (17).

Under these conditions, vaccination is increasingly considered a necessary complementary control strategy (21). However, this perspective becomes even more important as vaccination itself may further modify transmission dynamics and the selective landscape in which ASFV evolves.

## 4 Current status of ASF vaccines

ASFV has proven to be an exceptionally challenging target for vaccine development due to its complex genome, extensive immune evasion mechanisms, and the incomplete understanding of protective immunity (22). ASFV is a large, double-stranded DNA virus with a genome of approximately 170–190 kbp encoding more than 150 proteins. This genomic breadth exceeds that of many viral pathogens and includes numerous genes with immunomodulatory functions (23). Rather than encoding only structural components, ASFV dedicates a substantial portion of its genome to proteins that interfere with host immune mechanisms, including interferon signaling pathways, apoptosis regulation, antigen presentation, and macrophage activation (24).

ASFV exhibits a pronounced tropism for cells of the monocyte–macrophage lineage, which are central mediators of both innate and adaptive immunity. By replicating in and modulating these cells, the virus disrupts critical immune signaling cascades at an early stage of infection (25–27). This capacity for immune evasion and modulation complicates the identification of protective antigens and the definition of mechanisms underlying sterilizing immunity (21, 22). Consequently, a central obstacle in ASF vaccine development remains the absence of a clearly defined correlates of protection, which constrains the rational design, optimization, and comparative evaluation of candidate vaccines (28, 29).

Traditional vaccine platforms, including inactivated, subunit (30), vector-based (31), and multi-epitope vaccines (32) have so far failed to induce consistent and robust protection against ASF in experimental studies, or to demonstrate reliable protection in field settings. Chemically inactivated and irradiated vaccines have demonstrated the ability to stimulate immune responses and, in some cases, reduce viral load. However, they have not prevented the development of severe clinical disease following challenge with virulent ASFV strains (30, 33). Vaccinated animals frequently exhibited clinical outcomes comparable to unvaccinated controls. Consequently, despite extensive research efforts, conventional vaccine platforms have not yet provided a reliable solution, prompting a shift toward live attenuated approaches (20).

In light of the limited efficacy of inactivated and subunit approaches, current ASF vaccine development has primarily shifted toward live attenuated vaccines (LAVs) (8, 21, 34).

## 5 Live attenuated vaccines and safety

Attenuation is typically achieved through targeted deletions of virulence-associated genes, including members of multigene families involved in immune modulation (35, 36). However, the functional redundancy and incomplete characterization of many ASFV genes complicate predictions regarding long-term genetic stability (37). Experimental observations have reported prolonged viremia, mild or transient clinical signs, and concerns regarding genetic alterations after serial passage, underscoring the difficulty of ensuring stable attenuation under field conditions (20, 30, 34). Although several gene-deleted candidates have demonstrated protective efficacy against challenge strains, this strategy introduces distinct biological and epidemiological constraints. Because live attenuated vaccines (LAVs) are replication-competent, vaccinated animals may transiently harbour and potentially shed vaccine virus. This raises legitimate concerns regarding genetic stability, including risk of reversion to virulence, recombination with circulating field strains, persistence in tissues, and unintended transmission beyond target populations (29, 38). Reports from Asia describing genotype I/II recombinants and vaccine-related field isolates have reinforced the need for caution when considering the deployment of replication-competent vaccine strains in epidemiologically active settings (27, 39, 40). Importantly, ASFV genotype classification describes genetic relationships and should not be interpreted as a proxy for virulence, biological phenotype, or vaccine cross-protection. Consequently, genetic relatedness alone cannot be assumed to predict protection against emerging field variants. The ecological implications of such events extend beyond individual production systems as ASFV is maintained in wild boar populations, functioning as persistent and transboundary reservoirs (13, 41). The co-circulation of live attenuated vaccine strains and wild-type viruses in wild boars may increase the probability of vaccine–field recombinants with unpredictable phenotypic characteristics (13, 29). Thus, while live attenuated vaccines currently represent the most promising platform in terms of protective efficacy, their use requires rigorous regulatory oversight, structured field evaluation, and sustained post-vaccination monitoring to mitigate potential biological and epidemiological risks (20, 29). These considerations are reflected in recent WOAH recommendations for the field evaluation and post-vaccination monitoring of ASF vaccines, which emphasize the need to assess vaccine safety, effectiveness, genetic stability, and epidemiological consequences under field conditions (34).

An additional structural challenge in ASF vaccine development is the absence of widely validated and field-ready DIVA (Differentiating Infected from Vaccinated Animals) strategies (17). While some gene-deleted vaccine candidates incorporate molecular markers that may support differentiation under laboratory conditions (42, 43), no universally adopted and operationally robust DIVA framework currently exists. Without DIVA-compatible systems, vaccination campaigns risk interfering with international reporting obligations and trade transparency. For these reasons, the development of reliable differentiation strategies is not merely a technical refinement but a prerequisite for integrating vaccination into coherent ASF control frameworks (29).

## 6 Heterogeneity in surveillance sensitivity and data interpretation

Beyond vaccine design, the effectiveness and sustainability of any control strategy ultimately depends on the sensitivity, specificity, and comparability of surveillance systems. Under conditions of endemic persistence and wildlife-mediated transmission, surveillance sensitivity becomes a critical determinant of timely outbreak detection, epidemiological interpretation, and risk assessment (44, 45). Across Europe and parts of Asia, ASF surveillance systems differ substantially in sampling intensity, carcass search efforts in wild boar populations, diagnostic algorithms, and thresholds for initiating whole-genome sequencing (13, 18, 41, 45). In wild boar populations, early detection relies heavily on active carcass surveillance and timely laboratory confirmation. EFSA risk assessments have demonstrated that variability in carcass search effort and diagnostic capacity significantly influences apparent incidence and temporal detection patterns across Member States (13, 41). Consequently, differences in surveillance intensity may result in different epidemiological interpretations even under comparable underlying biological conditions.

In endemic settings characterized by circulation of strains associated with milder clinical presentations, reliance on overt mortality signals may further delay the identification of ongoing transmission (8). In the absence of systematic whole-genome sequencing, recombinant or vaccine-like variants may remain undetected (46, 47). However, in the absence of comparable surveillance sensitivity and harmonized investigative thresholds, emerging viral patterns may be recognized at different stages of dissemination, thereby complicating coordinated response efforts and transboundary risk assessment (29, 41).

## 7 Why coordination becomes critical

Taken together, the increasing evolutionary diversification of ASFV, the selective effects of modified control strategies, and the biological constraints of currently available vaccines indicate that ASF control has entered a structurally more complex phase. In this context, vaccination and surveillance cannot be understood as isolated technical measures within isolated production systems or confined national frameworks, rather, they are embedded in interconnected epidemiological systems whose stability depends on coordinated governance (3, 48). The evolving risk profile of the disease therefore reinforces the importance of existing international coordination mechanisms and shared standards, while highlighting the need to integrate vaccination policy, surveillance architecture, and evolutionary risk assessment more explicitly within transboundary ASF control framework (4, 41, 45, 49, 50).

Importantly, harmonization should not be mistaken for uniformity of implementation. Pig production systems differ fundamentally within and between regions, ranging from highly industrialized, vertically integrated enterprises to smallholder sectors shaped by chronic resource constraints, informal value chains, and limited access to veterinary services. In such contexts, biosecurity standards designed for capital-intensive systems may prove economically unrealistic or socially disruptive (51). As Chenais et al. (51) emphasize, persistent difficulties in smallholder settings reflect structural constraints that reinforce poverty–disease feedback loops. Coordination under these conditions thus demands shared epidemiological objectives, while allowing context-adapted implementation strategies supported by enabling structures, such as accessible animal health services, trusted local intermediaries, and value-chain incentives, that enhance feasibility and compliance across heterogeneous systems.

## 8 Conclusion: evidence points to collective responsibility

Accumulating evidence indicates that, in some regions with prolonged ASFV circulation, endemic persistence and viral diversification, coupled with adaptive responses to control measures, are becoming increasingly important determinants of the epidemiological landscape. Under these conditions, neither classical stamping-out strategies nor vaccination alone can be expected to restore a pre-epidemic equilibrium. Instead, ASF control must operate within a landscape shaped by ecological reservoirs, interconnected production systems, and ongoing viral evolution. In this context, vaccines represent an important technical advance and may substantially reduce mortality and production losses. However, they do not currently eliminate viral circulation, nor do they remove the biological constraints imposed by genetic variability and immune evasion. The potential deployment of LAVs introduces additional variables into an already dynamic system, reinforcing the need for rigorous field evaluation, structured monitoring, and transparent interpretation of emerging signals.

The central challenge is therefore not the absence of tools, but the necessity to apply them within a coherent, evidence-based framework that recognizes ASF as a long-term management problem rather than a short-term eradication campaign. In many regions, this implies a transition from eradication-oriented paradigms toward sustained adaptive risk management. Sustained data integration, comparability of surveillance outputs, and coordinated regulatory oversight are essential to preserve epidemiological clarity while adapting to evolving viral and production realities. In this context, ASF governance becomes an exercise in maintaining stability under conditions of persistent biological and structural complexity.

## Data Availability

The original contributions presented in the study are included in the article/supplementary material, further inquiries can be directed to the corresponding author.
